# Missed Opportunities for Preexposure Prophylaxis Initiation in Hospitalized Persons With Opioid Use Disorder and Infectious Diseases

**DOI:** 10.1093/ofid/ofae366

**Published:** 2024-07-10

**Authors:** Kaley Parchinski, Victor Neirinckx, Cynthia Frank, Angela Di Paola, Adati Tarfa, Sheela Shenoi, Brent Vander Wyk, Prerana Roth, Tracy Ghantous, Mary Kay Wegman, Michelle Strong, Frances R Levin, Kathleen Brady, Edward Nunes, Alain H Litwin, Sandra A Springer

**Affiliations:** Section of Infectious Diseases, Department of Internal Medicine, AIDS Program, Yale School of Medicine, New Haven, Connecticut, USA; Medical School, Medical College of Georgia, Augusta, Georgia, USA; Section of Infectious Diseases, Department of Internal Medicine, AIDS Program, Yale School of Medicine, New Haven, Connecticut, USA; Section of Infectious Diseases, Department of Internal Medicine, AIDS Program, Yale School of Medicine, New Haven, Connecticut, USA; Section of Infectious Diseases, Department of Internal Medicine, AIDS Program, Yale School of Medicine, New Haven, Connecticut, USA; Center for Interdisciplinary Research on AIDS, Yale University School of Public Health, New Haven, Connecticut, USA; Section of Infectious Diseases, Department of Internal Medicine, AIDS Program, Yale School of Medicine, New Haven, Connecticut, USA; Center for Interdisciplinary Research on AIDS, Yale University School of Public Health, New Haven, Connecticut, USA; Section of Infectious Diseases, Department of Internal Medicine, AIDS Program, Yale School of Medicine, New Haven, Connecticut, USA; Center for Interdisciplinary Research on AIDS, Yale University School of Public Health, New Haven, Connecticut, USA; Section of Geriatrics, Department of Internal Medicine, Yale School of Medicine, New Haven, Connecticut, USA; Department of Medicine, Prisma Health Addiction Medicine Center, Greenville, South Carolina, USA; Department of Medicine, University of South Carolina School of Medicine, Greenville, South Carolina, USA; Section of Infectious Diseases, Department of Internal Medicine, AIDS Program, Yale School of Medicine, New Haven, Connecticut, USA; Section of Infectious Diseases, Department of Internal Medicine, AIDS Program, Yale School of Medicine, New Haven, Connecticut, USA; Department of Medicine, Prisma Health Addiction Medicine Center, Greenville, South Carolina, USA; Department of Medicine, University of South Carolina School of Medicine, Greenville, South Carolina, USA; Department of Psychiatry, Columbia University Vagelos College of Physicians and Surgeons, New York City, New York, USA; Department of Psychiatry, New York State Psychiatric Institute, New York City, New York, USA; Department of Psychiatry, Medical University of South Carolina, Charleston, South Carolina, USA; Department of Psychiatry, Columbia University Vagelos College of Physicians and Surgeons, New York City, New York, USA; Department of Psychiatry, New York State Psychiatric Institute, New York City, New York, USA; Department of Medicine, Prisma Health Addiction Medicine Center, Greenville, South Carolina, USA; Department of Medicine, University of South Carolina School of Medicine, Greenville, South Carolina, USA; Section of Infectious Diseases, Department of Internal Medicine, AIDS Program, Yale School of Medicine, New Haven, Connecticut, USA; Center for Interdisciplinary Research on AIDS, Yale University School of Public Health, New Haven, Connecticut, USA

**Keywords:** HIV, opioid use disorder, preexposure prophylaxis, buprenorphine, infection, hospitalization

## Abstract

Hospitalizations are increasing among persons who use opioids, secondary to overdose and infections. Our study identified acute hospitalization as a reachable moment for engaging people who use drugs in increased screening and education about human immunodeficiency virus risk and prevention (preexposure prophylaxis).

## Teaser

In patients with opioid use disorder admitted to the hospital for an infection, less than half had heard of preexposure prophylaxis (PrEP) and none were taking PrEP. The majority of patients believed they had no or low risk of HIV infection.

A disproportionate number of people with opioid use disorder (OUD) also have human immunodeficiency virus (HIV). OUD can increase one’s risk of contracting HIV [1]. Currently about 1 in 10 new HIV diagnoses in the United States (US) can be attributed to injection drug use [2]. This increased risk may be related to using needles or other injection drug works contaminated with HIV but also engaging in risky sexual behaviors such as sex without a condom when under the influence of substances or exchanging sex for money or drugs [2-4]. People who inject drugs are also more likely to have multiple sexual partners as compared to those who do not inject drugs [3]. Therefore, this population needs HIV risk screening, education, and prevention interventions. One of the most effective HIV prevention strategies is preexposure prophylaxis (PrEP) [5].

Prior studies indicate a willingness to use PrEP among people who use drugs (PWUD) [6, 7]; unfortunately, there is little awareness of PrEP in this population [8, 9], and uptake is low due to myriad barriers ranging from cognitive (attitude, knowledge, internalized stigma, and societal stigma) to systemic (funding, advertising, and physician purview) [10].

Among persons with OUD, rates of hospitalization and emergency department visits are high [11, 12], and hospitalizations related to OUD with infectious diseases related to drug use via both injection and noninjection have increased in recent years [13].

Contact with the hospital healthcare system is an opportunity to identify PWUD, including those with OUD who are at risk of HIV, to provide HIV testing, assess for PrEP eligibility, and initiate PrEP. Hospital settings thus could be a key “reachable moment” to engage PWUD in HIV prevention and treatment to meet the Ending the HIV Epidemic goals [5, 14, 15].

This descriptive analysis sought to examine participants’ knowledge and attitudes about PrEP to better understand the potential of the acute medical hospitalization setting as an opportunity to increase PrEP uptake in participants with OUD. We hypothesized that participants would have a high knowledge of and interest in PrEP.

## METHODS

### Study Design and Population

Project COMMIT (Coordinated Medical Treatment of Opioid Use Disorder and Infectious Disease) is a National Center for Advancing Translational Sciences (U01TR002763) multisite randomized controlled trial of adults hospitalized with OUD and associated infections comparing integrated treatment of long-acting buprenorphine (Sublocade) with infectious disease care compared to treatment as usual [16]. For this analysis, we assessed PrEP knowledge and interest in a subpopulation of the parent COMMIT study. For the parent ongoing study, adults hospitalized with bacterial, fungal, and/or viral (eg, hepatitis C virus [HCV], HIV) infections who met criteria for a moderate to severe OUD, per *Diagnostic and Statistical Manual of Mental Disorders, Fifth Edition* (*DSM-5*) criteria, were recruited during hospital stays. After enrollment, baseline interview data were collected by study staff. If not available in the electronic health record, HIV and HCV testing was performed. For those with HIV and/or active HCV, viral load (VL) testing was obtained. The study was funded in July 2019, recruitment began in August of 2020 and ended in the spring of 2024 [16]. Primary outcome and key secondary outcomes of this parent study will be published separately. A detailed description of Project COMMIT's methodology has been previously published elsewhere [16].

Of the first 144 participants enrolled in the ongoing parent COMMIT trial, 75 participants completed a supplemental questionnaire assessing beliefs in PrEP. This questionnaire was added to the Yale New Haven Hospital site on 24 June 2021 and to other study sites on 12 April 2022. Two participants reported prior diagnosis of HIV and were therefore not eligible for PrEP and were excluded from analyses. Thus, 73 participants were included in this subanalysis with 40 participants recruited from Connecticut, 31 from South Carolina, and 2 from Pennsylvania. Of note, oral formulations of PrEP were available to patients at all hospital study sites. Injectable PrEP was not available at any of the study sites during the study.

### Statistical Analysis

Basic demographic information, sexual and HIV risk factors, and opioid use risk factors of the full sample, and beliefs about PrEP in a subsample, were reported as measures of central tendency, dispersion, and distribution. All descriptive analyses were performed using RStudio 2022.02.2 (RStudio, Inc, Boston, Massachusetts).

## RESULTS

Among the 73 participants included in this analysis, the mean age was 41.3 (standard deviation, 10.1) years. Of the 73, 36 (49.3%) identified as female, 60 (82.2%) were White, 8 (11.0%) were Hispanic, 34 (46.6%) were unhoused, 41 (56.2%) received a high school diploma/General Educational Development equivalent education or less, 31 (42.5%) were looking for a job or unemployed, 25 (34.2%) did not have health insurance coverage (including private, state, or federal health insurance), and 26 (35.6%) earned <$5000 annually.

Two participants (2.7%) had active hepatitis B infection (VL, 17.8 and 770 copies/mL, respectively), and 26 participants (35.6%) had active HCV infection (VL range, 18–14 700 000 copies/mL).

When evaluating reported HIV risk behaviors in the 30 days prior to hospitalization, 46 (63.0%) reported injection drug use, and 12 (16.4%) shared injection drug use works with unknown status partners. Approximately one-third of people surveyed knew their partners’ HIV status: 22 (30.1%) knew their partner was negative for HIV and 3 (4.1%) listed the partner status as HIV positive or unknown. Twenty-six participants (35.5%) reported condomless sexual intercourse in the last 30 days with unknown status partners.

### PrEP Questionnaire

Of 73 participants who completed the PrEP questionnaire, 29 (39.7%) had never heard of PrEP (Table 1). None had taken PrEP before. When asked about their perceived risk of HIV, on a 4-point scale (high, medium, low, or no risk), a majority of participants 64 (87.7%) thought they had no or low risk of HIV infection. Specifically, 41 of the 73 (56.2%) reported no risk of HIV infection.

**Table 1.** Results of a Preexposure Prophylaxis Questionnaire Administered to Hospitalized Persons With Opioid Use Disorder and Infectious Diseases

Question	No. (%) (N = 73^a^)
What do you think your current risk of contracting HIV is?	
No risk	41 (56.2)
Low risk	23 (31.5)
Medium risk	4 (5.5)
High risk	3 (4.1)
Missing	2 (2.7)
Have you ever heard of a medication for HIV preexposure prophylaxis known as PrEP?	
No	29 (39.7)
Yes	42 (57.5)
Missing	2 (2.7)
Are you currently taking PrEP? (n = 42)	
No	41 (97.6)
Yes	1 (2.4)
Have you ever taken PrEP before? (n = 41)	
No	41 (100.0)
Yes	0 (0)
Are you interested in taking PrEP now? (n = 72)	
No	60 (83.3)
Yes	10 (13.9)
Missing	2 (2.8)
Would you prefer a daily pill or a monthly injection? (n = 12)	
Daily pill	2 (16.7)
Monthly injection	9 (75.0)
Missing	1 (8.3)
Where would you prefer to get PrEP prescribed? (n = 12)	
Primary care physician	7 (58.3)
Emergency room	0 (0)
Substance use treatment program	1 (8.3)
Infectious diseases provider	1 (8.3)
Mobile health van	1 (8.3)
Telemedicine with physician (FaceTime/Zoom, etc)	0 (0)
Other	1 (8.3)
Missing	1 (8.3)
Why are you not interested in taking PrEP? (n = 60)	
Cost	0 (0)
Concern over side effects	0 (0)
Accessibility	0 (0)
I do not think I need it	54 (90.0)
I did not want to take the medicine	4 (6.7)
Other	2 (3.3)

Abbreviations: HIV, human immunodeficiency virus; PrEP, preexposure prophylaxis.

^a^Questions with a sample size other than 73 are noted and were part of a branched question response.

## DISCUSSION

In this secondary analysis of participants with OUD admitted to the hospital for infections related to drug use, a high proportion reported risky behaviors for HIV. However, only a minority were aware of PrEP, few saw the need for it, and only one had ever taken it. Almost half had never heard of PrEP.

Our findings illustrate missed opportunities for engagement in HIV prevention and are consistent with other studies that have found a low knowledge of PrEP among those with OUD and PWUD [8].

Persons with OUD stand to benefit from medications for OUD and PrEP initiation in hospital settings. Patients admitted to the hospital with OUD and a concomitant infection who do not have HIV are at risk for future HIV infection. Hospitalization is a crucial point of contact with the healthcare system where HIV testing can be offered along with assessment for PrEP eligibility and initiation of PrEP, then continued into the community. With the US Food and Drug Administration approval of a long-acting injectable PrEP (cabotegravir), providing PrEP during hospital admission for persons with OUD at risk for HIV could be even more convenient and could bridge the gap from inpatient to outpatient PrEP coverage. While cabotegravir efficacy at reducing injection risk has not been evaluated among PWUD, PWUD also should be assessed for condomless sexual intercourse risk and bacterial sexually transmitted infections, both of which are eligibility criteria for cabotegravir initiation. Furthermore, while a formal assessment of injectable PrEP to reduce injection risk has not been completed, one study found that the majority of people who inject drugs, when asked, stated injectable PrEP was acceptable [17].

Our findings, along with other previous studies that also found hospitalizations as a missed opportunity for HIV screening and PrEP initiation [14], should serve as a call to action for infectious disease clinicians and other hospital providers of PWUD. Previous work has outlined systems, tools, and treatment protocols for implementing hospital-based substance use care [15]. Infectious disease clinicians, hospitalists, addiction medicine/psychiatry, and other healthcare staff must lead the charge in implementation and be keenly aware of local substance use and HIV care resources to improve initiation and linkage to care for HIV prevention and treatment services postdischarge. Clinicians should also remember to assess sexual risk and PrEP eligibility among those who use/inject drugs. Additionally, tailored education and counseling specific to HIV risk factors and accessible educational materials are urgently needed. Targeted advertisements should be placed where persons with OUD who have minimal contact with the healthcare system may frequently visit, such as homeless shelters or harm reduction programs such as syringe service programs.

Although this study has a small sample size, limiting the types of analyses able to be conducted, the findings highlight missed opportunities for improving care for PWUD. Additionally, future studies could be aimed at characterizing why those who wanted to initiate PrEP at discharge were not started on PrEP.

## CONCLUSIONS

This study identifies the need to strengthen education and awareness about PrEP among PWUD and attention to acute hospitalization as a reachable moment for engaging them in HIV prevention care. Both are imperative to increasing PrEP uptake among those at high risk for future HIV infection and are essential to achieving the Ending the HIV Epidemic goals.

## Notes

***Author contributions.*** K. P. and S. A. S. were responsible for the conceptualization, methodology, and visualization. K. P., V. N., B. V. W., T. G., M. K. W., C. F., and A. d. were responsible for data curation. K. P., S. S., and S. A. S. wrote the original draft. K. P. conducted the formal analysis. A. T., S. S., P. R., M. S., F. R. L., K. B., E. N., A. H. L., and S. A. S. provided writing, reviewing, and editing. S. A. S. received the funding and directed the study the paper is based on. All authors read and approved the final manuscript.

***Patient consent.*** All protocols and study procedures were approved by the institutions’ internal review boards and the Office of Human Research Protections at the Department of Health and Human Services. A Certificate of Confidentiality was obtained for additional participant protections. The study was registered at www.clinicaltrials.gov (NCT04180020).

***Disclaimer.*** The funders were not involved in the research design, analysis or interpretation of the data, or the decision to publish the manuscript.

***Data availability.*** Data are not currently publicly available but will be upon publication of the parent study.

***Financial support*.** This work was supported by the National Center for Advancing Translational Sciences (NCATS; grant number U01TR002763 to S. A. S., K. B., F. R. L., and E. N.); National Institute on Drug Abuse (NIDA; grant number DP1DA056106 to S. A. S.); and Claude D. Pepper Older Americans Independence Center from the National Institute on Aging (grant number P30AG021342 to B. V. W.).
